# Intestinal Microbiota Play an Important Role in the Treatment of Type I Diabetes in Mice With BefA Protein

**DOI:** 10.3389/fcimb.2021.719542

**Published:** 2021-09-17

**Authors:** Qi Qin, Yan Chen, Yongbo Li, Jing Wei, Xiaoting Zhou, Fuyin Le, Hong Hu, Tingtao Chen

**Affiliations:** ^1^National Engineering Research Center for Bioengineering Drugs and the Technologies, Institute of Translational Medicine, Nanchang University, Nanchang, China; ^2^Harbin Meihua Biotechnology Co., Ltd, Research and Development Center, Haerbin, China; ^3^School of Life Sciences, Lanzhou University, Lanzhou, China; ^4^Department of Dialysis, Haifushan Hospital, Weifang, China; ^5^Department of Orthopedics, Haifushan Hospital, Weifang, China; ^6^School of Life Sciences, Nanchang University, Nanchang, China; ^7^Center for Reproductive Medicine, Qingyuan Peopler’s Hospital, The Sixth Affiliated Hospital of Guangzhou Medical University, Qingyuan, China

**Keywords:** T1DM, BefA protein, inflammation, intestinal barrier, intestinal microbiota

## Abstract

More and more studies have shown that the intestinal microbiota is the main factor in the pathogenesis of type 1 diabetes mellitus (T1DM). Beta cell expansion factor A (BefA) is a protein expressed by intestinal microorganisms. It has been proven to promote the proliferation of β-cells and has broad application prospects. However, as an intestinal protein, there have not been studies and reports on its application in diabetes and its mechanism of action. In this study, a T1DM model induced by multiple low-dose STZ (MLD-STZ) injections was established, and BefA protein was administered to explore its therapeutic effect in T1DM and the potential mechanism of intestinal microbiota. BefA protein significantly reduced the blood glucose, maintained the body weight, and improved the glucose tolerance of the mice. At the same time, the BefA protein significantly increased the expression of ZO-1, Occludin, and significantly reduced the expression of TLR-4, Myd88, and p-p65/p65. BefA protein significantly reduced the relative expression of pro-inflammatory cytokines IL-1β, IL-6 and TNF-α. In addition, our high-throughput sequencing shows for the first time that the good hypoglycemic effect of BefA protein is strongly related to the increase in the abundance of the beneficial gut bacteria *Lactobacillus*, *Bifidobacterium* and *Oscillospria* and the decrease in the abundance of the opportunistic pathogen *Acinetobacter*. Our group used animal models to verify the hypoglycemic effect of BefA protein, and first explored the potential mechanism of intestinal microbiota in BefA protein treatment.

## Introduction

Diabetes (Diabetes Mellitus, DM) is a group of metabolic diseases that chronically increase blood glucose levels due to defective insulin secretion or impaired biological effects, and is mainly divided into type 1 (insulin-dependent, T1DM) diabetes and type 2 (non-insulin-dependent, T2DM) diabetes. T1DM is characterized by autoimmune damage of pancreatic islet β cells and absolute insufficient insulin secretion. Most of the patients are under 30 years old, the number of patients accounts for about 5%-10% of all patients with diabetes, and it is expected to increase rapidly at a rate of 3% every year (Maffi and Secchi, 2017; Saeedi et al., 2019). T1DM has a relatively rapid onset, with obvious symptoms of polydipsia, polyuria, polyphagia and weight loss, and when the disease gets worse, it will cause systemic metabolic disorders (acidosis) in the body (Katsarou et al., 2017). In addition, long-term hyperglycemia in patients with T1DM is also a major factor leading to chronic damage and dysfunction of various tissues such as liver, kidney, heart, retina, and cerebrovascular (Dimeglio et al., 2018). Among them, diabetic heart disease, cerebrovascular illness and kidney disease are the main reasons of death, bringing a heavy burden to the patient’s family and social medical system (Deshpande et al., 2008; Núñez et al., 2019).

Due to the destruction of pancreatic β-cells lead to an absolute lack of insulin secretion, subcutaneous injection of insulin has been the most important treatment for T1DM (Warshauer et al., 2020). In recent years, with the widespread application of insulin sustained-release pump technology and genetically engineered long-acting insulin, the blood sugar of T1DM patients has been more effectively controlled than before (Beck et al., 2019). However, the insulin injected into the body cannot be regulated by blood sugar, and the release of blood sugar does not conform to the circadian rhythm, patients’ blood sugar often fluctuates greatly, which can easily cause hypoglycemia and syncope (Allen and Frier, 2003). Although type 2 diabetes treatment drugs for instance biguanides, α-glycosidase inhibitors, thiazolidinediones, sodium-dependent glucose transporter 2 (SGLT-2) inhibitors, dipeptidyl peptidase-4 (DPP-4) inhibitors and glucagon-like peptide 1 (GLP-1) receptor agonists (Mehanna, 2013; Clapham, 2020) are gradually used in adjuvant treatment of T1DM (Warnes et al., 2018). However, each of the above drugs has drawbacks: biguanide drugs lead to stomach discomfort or anorexia easily. Individuals may also suffer lactic acidosis or ketoacidosis, which can reduce the absorption of vitamin B12 in intestines and decline hemoglobin synthesis, leading to macrocytic anemia, even malabsorption (Defronzo et al., 2016; Mccreight et al., 2016); Alpha-glycosidase inhibitors account for gastrointestinal side effects including bloating, abdominal pain, diarrhea, and gastrointestinal cramps (Aoki et al., 2010); Thiazolidinedione hypoglycemic drugs have important advantages for patients in obvious insulin resistance, but they will give rise to weight gain, fluid retention, and increase the risk of heart failure (Kung and Henry, 2012); SGLT-2 inhibitors lower the blood sugar by increasing urine sugar secretion, leading to the risk of genitourinary system infections, fractures and lower limb amputations, and increased risk of ketoacidosis (Halimi and Vergès, 2014); GLP-1 receptor agonists and DPP-4 inhibitors are expensive and require injections. Although it is reported that pancreatic islet transplantation or stem cell technology can cure T1DM, it is still in the experimental stage due to insufficient donors, transplant immune rejection, and the potential carcinogenic risk of stem cells (Wu and Mahato, 2014; Dean et al., 2017). Therefore, it is of vital significance to develop and study T1DM drugs with small side effects, lower economic costs, and oral administration!

The gastrointestinal tract of mammals has a large number of different types and numbers of microorganisms, called the gut microbiota. With the rapid development of microbial detection technology, more and more evidences show that the intestinal microbiota plays a vital role in a variety of metabolic diseases (diabetes, tumors, gastroenteritis, nervous system diseases, *etc*.) (Heintz-Buschart et al., 2016; De Vadder et al., 2018; Simrén, 2018; Riquelme et al., 2019), especially the relationship between diabetes and gut microbes has become one of the current research hotspots. A study in December 2016 showed that Beta Cell Expansion Factor A (BefA), a gut bacterial protein newly discovered by researchers at the University of Oregon, could induce the proliferation of insulin-producing β cells in the pancreas during the early development of zebrafish larvae (Hill et al., 2016). This research is of great significance to human health, because β cells are the only cells that produce insulin, and insulin is responsible for regulating blood sugar metabolism, which is closely relative to diabetes. Most importantly, the study not only confirmed that microbiota affects the development of β cells in animals in the zebrafish model, but also found that BefA protein also exists in several common human intestinal-related bacteria. Moreover, the researchers found that two related proteins purified from human bacteria also effectively promoted the proliferation of β-cells in zebrafish, suggesting that BefA protein may promote β cells proliferation in other animals, including humans (Hill et al., 2016). This gratifying discovery provides new possibilities for the treatment of diabetes.

BefA protein is derived from the intestinal microbiota and promote the proliferation of pancreatic β-cells in zebrafish, which is of great significance for the treatment of human diabetes. However, the therapeutic effect and mechanism of BefA protein in mammalian diabetes is still unknown. Therefore, a T1DM model of C57BL/6J mice was developed through MLD-STZ injections and treated with BefA purified protein. For the first time, the therapeutic effect of BefA protein in T1DM and the potential mechanism of intestinal microbiota in the treatment were explored.

## Materials and Methods

### Animal Studies

Forty-eight eight-week-old female C57BL/6J mice (20-22g), provided by Hunan SJA Laboratory Animal, were maintained in the specific pathogen free (SPF) laboratory animal barrier system of the Institute of Translational Medicine of Nanchang University under standard conditions (humidity 51 ± 13%, temperature 23 ± 3°C, 12/12 light-dark cycle) and were fed with standard mice maintain diet (Xietong biological, CN, Cat# 101139).

Then, all mice were randomly divided into four groups: (1) Control group (C, n=12): oral administration of 100μL coating solution (0.01% gelatin dissolved in saline) per two day until the end of the experiment; (2) Model group (M, n=12): mice were intraperitoneally (i.p.) injected with 40mg/kg STZ (dissolved in 0.1M citrate buffer, pH=4.5) for five consecutive days, then oral administration of 100μL coating solution per two day until the end of the experiment after the blood sugar stabilizes. (3) BefA protein treatment group (MB, n=12): mice were i.p. injected with 40mg/kg STZ for five consecutive days, then oral administration of 2mg/kg BefA protein (dissolved in the coating solution) per two day until the end of the experiment after the blood sugar stabilizes. (4) Metformin protein treatment group (MM, n=12): mice were i.p. injected with 40mg/kg STZ for five consecutive days, then oral administration of 0.2g/kg metformin (dissolved in the coating solution) per two day until the end of the experiment after the blood sugar stabilizes.

At day 28^th^, fecal samples were collected, all mice were anesthetized with 1% sodium pentobarbital solution, and the cervical spine was dislocated to death. after glucose tolerance test, and the colon was removed and frozen at -80°C for further use.

### Construction of the BefA Yield Strain and Protein Purification

The BefA gene (M001_10165) was codon optimized for *Escherichia coli* BL21 to favor higher protein yield, and was synthesized with a histidine (His) tag (to facilitate the identification and purification of BefA protein), which was inserted into the prokaryotic expression vector pet 28C in Kingsy Biotechnology Co. (Nangjing, China) to form the recombinant plasmid pet 28C-BefA. Then, pet 28C-BefA was transformed into *Escherichia coli* BL21 strain to generate the BefA production strain of BL21-pet 28C-BefA.

To produce BefA protein, the BL21-pet 28C-BefA strain was cultivated in Luria-Bertani (LB) medium (Solarbio Life Sciences, China, Cat# L1010) with kanamycin (50 μg/ml. Solarbio Life Sciences, China, Cat# K1030) at 37°C. When the optical density value reached to 0.6-0.8, 1 mM of isopropyl β-D-thiogalactoside (IPTG, Solarbio Life Sciences, China, Cat# I8070) was added into culture medium to stimulate massive protein expression during the following 6-h cultivation. Then the culture medium was centrifuged at 8,000 g for 30 min to obtain the bacterial pellet, which was further used for ultrasonic disintegration to flow out bacterial proteins. BefA protein was purified with His-tag nickel beads (7Sea Biotech, China, Cat# PAN001-001C), and the purity and accuracy of BefA protein were detected by SDS-PAGE electrophoresis and Western-blotting, and the purified protein concentration was determined by BCA protein assay kit (Thermo Fisher, USA, Cat# 23227) according to the manufacturers guidelines.

### Glucose Tolerance Test

GTT analyses were performed in C57BL/6J mice at the end of experiment. The mice were fasted for 16 h before the test. Blood samples from the tail vein were collected at 0, 15, 30, 60, and 120 minutes after i.p. injection of 2g/kg glucose, and the glucose concentration was measured with a blood glucose meter.

### Western Blotting

The colon tissue was taken into centrifuge tube, tissues were homogenized on ice after adding proper amount of RIPA lysis buffer (Solarbio, CN, Cat# R0010) mixed with protease inhibitors cocktail (Thermo Fisher, USA, Cat# 78429). The supernatant was obtained by centrifugation at 12000g for 10 min at 4°C, and the protein concentration was determined. Then, the protein was separated on 10%~12% sodium dodecyl sulfate-polyacrylamide gel electrophoresis (SDS-PAGE) and transferred to polyvinylidene fluoride (PVDF) membrane. Blocked in 5% skim milk-TBST [20 mM Tris-HCl (pH 7.6), 127 mM NaCl, 0.1% Tween 20] at room temperature for 2 h. After washing with TBST, incubate the PVDF membrane with appropriately diluted primary antibody at 4°C overnight. After washing again with TBST, incubate the membrane with 1% skim milk-TBST diluted secondary antibody at room temperature for 90 minutes. The following antibodies were used: rabbit anti- tight junction protein 1 (zona occludens 1, ZO-1; 1:5000; Proteintech; Cat# 21773-1-AP), rabbit anti- Occludin (Occludin, 1:1000; Proteintech; Cat# 13409-1-AP), rabbit anti- β-actin (1:1000; Cell Signaling Technology, Cat# 4970S), mouse anti- Toll-like receptor 4 (TLR4, 1:1000; Santa Cruz Biotechnology, Cat# sc-293072), rabbit anti- myeloid differentiation primary response gene (88) (MyD88, 1:1000; Proteintech; Cat# 23230-1-AP), rabbit anti- p65 (1:1000; Cell Signaling Technology, Cat# 8242S), rabbit anti- p-p65 (1:1000; Cell Signaling Technology, Cat# 3031S).

### Gene Expression Analysis

The frozen colon tissue in liquid nitrogen was fully grinded and added with TRIzol reagent (Thermo Fisher, USA, Cat# 15596018) to extract total RNA. Use RNase-free DNase І (Takara Bio, JP, Cat# RR047A) to remove genomic DNA, and a NanoDrop 2000 spectrophotometer (Thermo Fisher, Massachusetts, USA) was used to determine the concentration and quality of extracted RNA. According to the manufacturer’s instructions, PrimeScript™ RT Master Mix (Takara Bio, JP, Cat# RR036Q) was used to generate complementary DNA (cDNA). The 7900HT fast real-time PCR system (ABI, Foster City, CA) of 2×SYBR Green master Mix was performed to quantify gene expression and normalized with reference to the gene glyceraldehyde 3-phosphate dehydrogenase (GAPDH).

The sequences of the primers are as follows: IL-1β (forward_5′-GTGTCTTCCCGTGGACCTTC-3′, reverse_5′-TCATCTCGGAGCCTGTAGTGC-3′), IL-6 (forward_5′-GAAATCGTGGAAATGAG-3′, reverse_5′-GCTTAGGCATAACGCACT-3′), TNF-α (forward_5′-GTGGAACTGGCAGAAGAGGCA-3′, reverse_5′-AGAGGGAGGCCATTTGGGAAC-3′) and GAPDH (forward_5′-CTCGTGGAGTCTACTGGTGT-3′, reverse_5′-GTCATCATACTTGGCAGGTT-3′).

### DNA Extraction and Bacterial 16S rDNA Sequencing

Collect faecal samples from groups C (N = 5), M (N = 5), MB (N = 5) and MM (N = 5). According to the manufacturer’s instructions, using TIANamp bacterial DNA kit (TianGen, CN, Cat# DP302) to extract bacterial genomic DNA. A NanoDrop spectrophotometer was used to determine the concentration and quality of the extracted DNA. Use primers 515F (5’-GTGCCAGCMGCCGCGGTAA-3’) and 806R (5’-GGACTACVSGGGTATCTAA T-3’) to amplify 16S ribosomal DNA (rDNA) V4 region, and the PCR products were sequenced on the IlluminaHiSeq 2000 platform (Illumina, Inc, San Diego, CA).

### High-Throughput Sequencing Analyses

Paired-end sequencing was performed on community DNA fragments using the Illumina platform (version 2019.4, https://docs.qiime2.org/2019.4/tutorials/) [18]. First call qiime cutadapt trim-paired to remove the sequence of primer fragments, discard the sequence of unmatched primers; then call DADA2 for quality control, denoising, splicing and de-chimerism through qiime dada2 denoise-paired. After denoising all libraries, merge the ASVs feature sequence and ASV table, and remove the singletons ASVs. After obtaining the ASV characteristic sequence or the OTU representative sequence, the length distribution of the high-quality sequence contained in the sample is counted. According to the distribution of ASV/OTU in different samples, the diversity level of each sample was evaluated, and the applicability of sequencing depth was reflected through the sparse curve. At the ASV/OTU level, calculate the distance matrix of each sample, and measure the diversity and difference between different samples (groups) through various unsupervised sorting and clustering methods combined with corresponding statistical testing methods. High-throughput sequencing data has been uploaded to NCBI, GenBank accession number PRJNA664289.

### Statistical Analyses

Data analyses were performed by Prism 7 (GraphPad, San Diego, USA). One- or two-way analysis of variance (ANOVA) followed by Tukey’s multiple comparison test was used in all studies, as noted in figure legends. Data are presented as mean ± standard deviation (SD). Statistical significance was defined as *p < 0.05, **, *p* < 0.01.

## Results

### BefA Protein Improves Blood Glucose and Body Weight in MLD-STZ Induction of T1DM Mice

**Figure 1:**
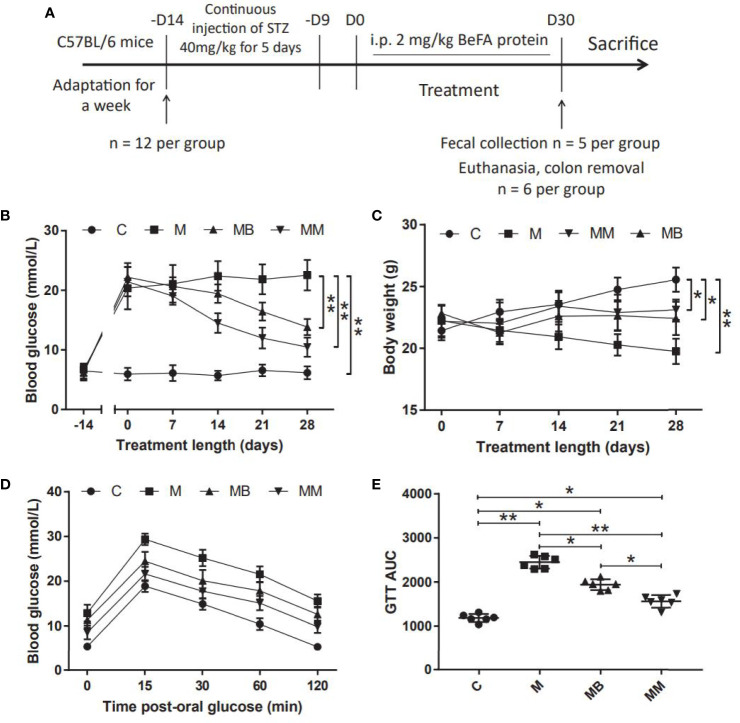
BefA protein improves blood glucose and body weight in MLD-STZ induction of T1DM mice. **(A)** Treatment schedule of BefA protein for MLD-STZ induction of T1DM model. **(B, C)** Changes in blood sugar and body weight over time in C, M, MB and MM group (n = 12). **(D, E)** Levels of blood glucose and area under the curve (AUC) during a GTT in C, M, MB and MM group (n = 6). Data are presented as means ± SD. Two-way repeated-measures ANOVA both with Tukey’s test for multiple comparisons (**B–D**, respectively) and One way repeated-measures ANOVA with Tukey’s test for multiple comparisons **(E)**; *p < 0.05, **p < 0.01.

A mice model of T1DM induced by MLD-STZ was established to evaluate the therapeutic effect of BefA protein and the potential mechanism of intestinal microbiota in the treatment (**Figure 1A**). Our results showed that the BefA protein showed a good hypoglycemic effect from the 14th day, and the 28th day had a more significant hypoglycemic effect compared with the M group (13.84 mmol/L *vs* 22.52 mmol/L, *p* < 0.01) (**Figure 1B**). The weight measurement results showed that the weight of the mice in the M group decreased over time, while the C group had the opposite weight gain. Similar to the therapeutic effect of metformin, BefA protein maintain the weight of mice and prevent weight loss (**Figure 1C**). In addition, the GTT results showed that, the BefA protein significantly improved the blood glucose tolerance of the mice compared with the M group. The area under curve (AUC) of glucose analysis also had similar results. The M group possessed the largest AUC compared to MB group (2450 *vs.* 1940.5, *p* < 0.05) or MM group (2450 *vs.* 1561, *p* < 0.01) (**Figures 1D, E**).

### Befa Protein Reduced Intestinal Inflammation and Improved Intestinal Permeability

**Figure 2:**
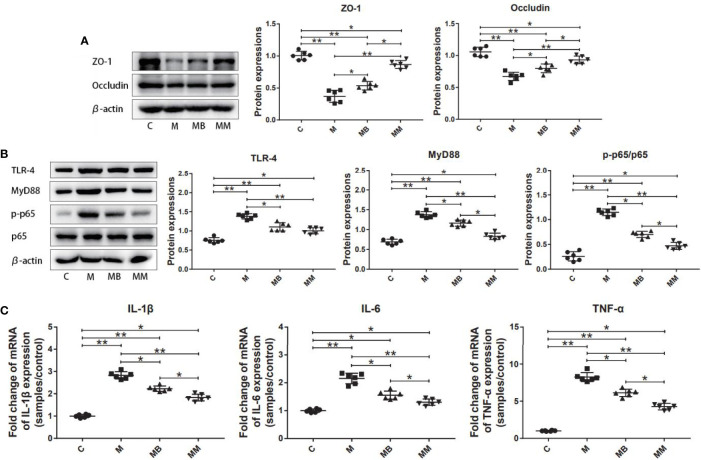
BefA protein reduced intestinal inflammation and improved intestinal permeability. **(A)** Western blot analysis of ZO-1 and Occludin expression in colon tissues (n = 6), β-actin was used as an internal control. The relative expressions of ZO-1 and Occludin were quantified by Image J. **(B)** Western blot analysis of TLR-4, MyD88, p-p65 and p65 expression in colon tissues (n = 6), β-actin was used as an internal control. The relative expressions of TLR-4, MyD88, p-p65 and p65 were quantified by Image J. **(C)** Pro-inflammatory cytokines IL-1β, IL-6 and TNF-α were detected in colon tissues at gene level by q-PCR (n = 6). Data are presented as means ± SD. One way repeated-measures ANOVA with Tukey’s test for multiple comparisons (**A–C**, respectively); *p < 0.05, **p < 0.01.

To explore the influence of BefA protein on the intestinal tissue, the key proteins and genes in the intestinal permeability and inflammation pathway (NFκB signal transduction) were further studied. As shown in **Figure 2**, treatment with BefA protein and metformin significantly increased the expression of ZO-1 (0.54 and 0.87, respectively) and Occludin (0.80 and 0.93, respectively), while significantly reducing the expression of TLR-4 (1.12 and 1.00, respectively), Myd88 (1.16 and 0.83, respectively) and p-p65/p65 (0.70 and 0.47, respectively) compared with M group (**Figures 2A, B**; *p* < 0.05). In addition, BefA protein and metformin significantly reduced the relative expression of pro-inflammatory cytokines IL-1β (2.23 and 1.83, respectively), IL-6 (1.56 and 1.31, respectively) and TNF-α (6.13 and 4.27, respectively) compared with M group (**Figure 2C**; *p* < 0.05).

### The Influence of BefA Protein on Intestinal Microbiota

**Figure 3:**
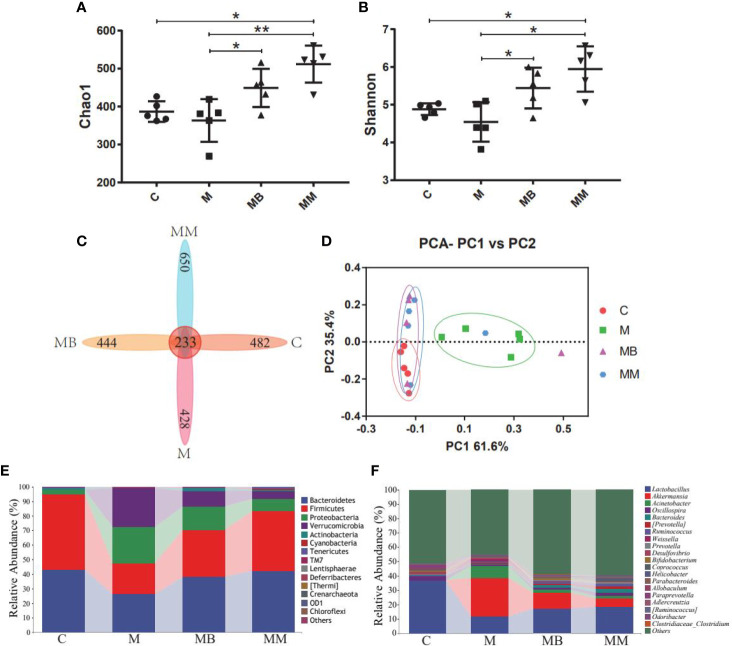
BefA protein on intestinal microbiota of the T1DM model. **(A)** the Chao1 index. **(B)** the Shannon index. **(C)** Venn map representation of OTUs. **(D)** PCA of β diversity index. **(E)** Microbial composition at the phylum level. **(F)** Microbial composition at the genus level. Data are presented as means ± SD. One way repeated-measures ANOVA with Tukey’s test for multiple comparisons (**A, B**, respectively); *p < 0.05, **p < 0.01.

High-throughput sequencing was used to study the effect of BefA protein on the intestinal microbiota of type I diabetic mice. A total of 1,244,702 filtered clean tags (62,235 tags/sample) and 8,115 OTUs were obtained from all samples, with an average of 2,029 OTUs per group (data not shown). In order to further analyze the effect of BefA protein on the intestinal microbiota of mice, the Chao1 index (evaluating community diversity) and Shannon index (estimating the total species) of α diversity analysis were carried out. The results showed that there was no significant change in C group and M group, while treatment with BefA protein and metformin significantly increased microbial diversity and microbial abundance (**Figures 3A, B**). When analyzed by the Venn method, 233 common OTUs were identified from all groups, and the unique OTU numbers in the C, M, MB, and MM groups were 482, 428, 444, and 650, respectively (**Figure 3C**). PCA analysis showed that the spots in group C were clustered, group M was relatively scattered, while the distribution of samples in group MB and MM were similar, and partially overlapped with group C, indicating that the microbial diversity of group MB and MM had high similarity with group C (**Figure 3D**).

The relative abundance of the top ten microorganism populations indicated that Bacteroidetes, Firmicutes, Proteobacteria constituted the three most common and dominant phyla in the C (0.43 *vs.* 0.52 *vs.* 0.03), M (0.26 *vs.* 0.21 *vs.* 0.25), MB (0.38 *vs.* 0.32 *vs.* 0.16) and MM (0.42 *vs.* 0.41 *vs.* 0.08). Combined, these phyla accounted for 97.82%, 72.33%, 86.59% and 91.67%, respectively, of the total sequencing number in these 4 groups (**Figure 3E**). At the genus level, data of top 20 microbial populations was analyzed. As shown in **Figure 3F**, *Lactobacillus*, *Akkermansia*, *Acinetobacter* and *Oscillospira* constituted four common dominant genus in C group (0.3673 *vs.* 0.0004 *vs.* 0.0009 *vs.* 0.0287), M group (0.1180 *vs.* 0.2677 *vs.* 0.0872 *vs.* 0.0052), MB group (0.1725 *vs.* 0.1077 *vs.* 0.0228 *vs.* 0.0162) and MM group (0.1857 *vs.* 0.0583 *vs.* 0.0142 *vs.* 0.0253). Among them, *Lactobacillus* dominates the C, MB and MM groups, and the relative abundance of *Akkermansia* in the M group is significantly higher than that in the C group.

**Figure 4:**
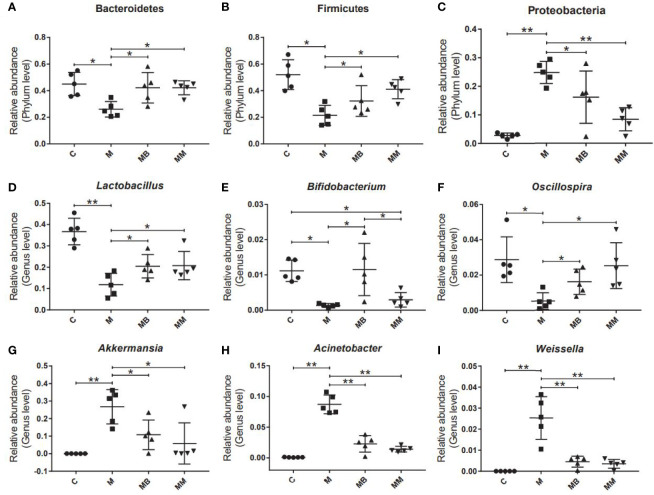
BefA protein regulated blood sugar by increasing the abundance of intestinal beneficial bacteria. The relative abundance of Bacteroides **(A)**, Firmicutes **(B)**, Proteobacteria **(C)**, *Lactobacillus* **(D)**, *Bifidobacterium* **(E)**, *Oscillospria* **(F)**, *Akkermansia* **(G)**, *Acinetobacter* **(H)** and *Weissella* **(I)** were analyzed. Data are presented as means ± SD. One way repeated-measures ANOVA with Tukey’s test for multiple comparisons (**A–I**, respectively); *p < 0.05, **p < 0.01.

Finally, some typical bacteria closely related to diabetes were selected. The results showed that the diabetes model significantly increased the relative abundance of Proteobacteria, *Akkermansia*, *Acinetobacter* and *Weissella* (**Figures 4C, G-I**). Compared with the M group, BefA and metformin treatment significantly increased the relative abundance of Bacteroides, Firmicutes, *Lactobacillus* and *Oscillospira* (**Figures 4A, B, D, F**) and reduced the relative abundance of Proteobacteria, *Akkermansia*, *Acinetobacter* and *Weissella* (**Figures 4C, G–I**). In addition, the BefA protein significantly increased the relative abundance of the beneficial intestinal bacteria *Bifidobacterium* compared to the M and MM group (**Figure 4E**).

## Discussion

The global incidence of T1DM is rising. Every year, more than 110,000 children and adolescents under the age of 15 are diagnosed with T1DM, imposing a heavy economic burden on society and families (Cho et al., 2018). Insulin, as the main drug for regulating blood sugar in patients with T1DM, can easily lead to drug resistance in patients with long-term use. Moreover, the dose of insulin tends to increase as the disease progresses, which also leads to weight gain and increased cardiovascular risk in patients (Kamal et al., 2006). Although real-time blood glucose monitoring systems, automated insulin delivery equipment (artificial pancreatic islets), pancreas transplantation and cell therapy were developed to treat T1DM, their applications are limited due to supply constraints (based on donors) and the need for chronic immunosuppression (El-Khatib et al., 2017; Shapiro et al., 2017; Weisman et al., 2017). In addition to insulin, the Food and Drug Administration (FDA) only approved pramlintide for T1DM, and there is still a lack of effective treatments for T1DM. Although in theory there are still many studies on auxiliary blood glucose regulation drugs that have not been recommended by the guidelines and have not been used on a large scale, they still provide new options and hopes for the treatment of patients with T1DM.

With the development of molecular biology technology, people have been able to more deeply explore the influence of intestinal microbiota on T1DM. The latest mice study on Cell Metabolism found that specific metabolites produced by the intestinal microbiota can act on the natural lymphocytes of the pancreas to promote the expression of β-defensin 14 in pancreatic endocrine cells, thereby preventing T1DM (Miani et al., 2018). This study revealed the pathogenesis of this type of diabetes and provided a new target for the treatment of T1DM. The BefA protein is also a protein expressed by intestinal microbes. It has been confirmed by Professor Guillemin that it can promote the proliferation of β cells and has broad application prospects (Hill et al., 2016). However, as an intestinal protein, there is no research report on its application in mammalian diabetes. Therefore, this study is the first to study the therapeutic effect of BefA protein on T1DM and its mechanism of action by establishing MLD-STZ induced T1DM models, which provides data support for the clinical application of BefA protein.

Consistent with previous studies, STZ selectively destroyed pancreatic islet β cells, causing increased blood sugar in mice in group M, while treatment with BefA protein and metformin for 4 weeks significantly reduced blood sugar (Sakata et al., 2012). In addition, the pathogenesis of T1DM was accompanied by typical “Three more and one less” symptoms (polydipsia, polyphagia, polyuria, weight loss), the weight of mice in the M group continued to drop, while BefA protein and metformin maintained the weight of the mice. Consistent with our expected results, the glucose tolerance test also confirmed that BefA protein and metformin can significantly improve the glucose tolerance of diabetic mice. Therefore, BefA protein treatment can indeed reverse blood glucose in mice.

T1DM is a chronic pro-inflammatory autoimmune disease caused by various risk factors (including genetics, chemicals, viruses, symbiotic bacteria, and diet) (Atkinson and Eisenbarth, 2001). In recent years, the intestinal microbiota has been considered as the important factor in the pathogenesis of T1DM. The intestinal microbiota directly interacts with the adjacent mucosal environment, affecting intestinal permeability, and affecting local and systemic inflammatory activities (Needell and Zipris, 2016; Zheng et al., 2018).

Therefore, in order to further study the potential mechanism of BefA protein in the treatment of diabetes, we have studied the key proteins in the pathways related to inflammation and intestinal permeability. As we all know, the intestinal barrier can effectively protect the body and prevent harmful substances and pathogens from entering the blood circulation and other tissues and organs. Tight junction proteins such as Occludin and ZOs play an important role in the maintenance of the intestinal mucosal epithelial mechanical barrier. By connecting epithelial cells, tight junction proteins close the gaps between cells to maintain cell polarity. (Limit the free diffusion of lipids and intact membrane proteins between different cell liquids) and permeability barriers (only allow ions and soluble small molecules to pass, and regulate the passive transport of ions and macromolecular substances across the cell bypass) (Chelakkot et al., 2018; Chen et al., 2020). Down-regulation of its expression or decreased activity will affect the formation of tight junctions between cells, hinder the intestinal mucosa from exerting its important defense barrier function, and increase the risk of intestinal infection caused by harmful bacteria and toxins penetrating the intestine into the bloodstream (Slifer and Blikslager, 2020). NF-κB is an important factor in the immune and inflammatory process of the body. After the intestinal microbiota is unbalanced, the tight junction complex composed of transmembrane proteins (Occludin, Claudins, *etc*.), binding proteins (ZO-1, pl30, *etc*.) and cytoskeleton structure is destroyed, resulting in increased intestinal wall permeability. The impaired intestinal barrier can cause the translocation of infectious factors such as lipopolysaccharide (LPS) and food antigens into the blood, promote various Toll-like receptors to activate NF-κB, further stimulate the massive release of pro-inflammatory factors (IL-1β, IL-6, TNF-α, *etc*.), and inducing inflammation and insulin resistance of liver, muscle and adipose tissue, leading to the occurrence of type I and type II diabetes (Luissint et al., 2016; Tilg et al., 2020). Therefore, the significant increase of ZO-1 and Occludin, the significant decrease of TLR-4, MyD88, pp65/p65, IL-1β, IL-6 and TNF-α indicate that BefA protein can significantly improve intestinal permeability and reduce intestinal and Inflammation of the body (**Figure 2**).

Finally, a high-throughput sequencing method is used to monitor the changes of the host’s gut microbes. Compared with mice in the M group, treatment with BefA and metformin increased the diversity and abundance of microorganisms, and promoted the transformation of the intestinal microbiota of diabetic mice to the control group. Studies have shown that the intestinal microbes of healthy mice are maintained in a normal equilibrium state, with Bacteroides, Firmicutes and Proteobacteria accounting for the highest relative abundance. The imbalance of the microbiota (increased abundance of Proteobacteria, decreased abundance of Bacteroides and Firmicutes) is highly related to the occurrence and development of intestinal diseases, diabetes and other metabolic diseases (Ho et al., 2020; Tilg et al., 2020). Our research found that BefA and Metformin can restore the balance of intestinal microbiota by increasing the relative abundance of Bacteroides and Firmicutes and inhibiting the relative abundance of Proteobacteria. In addition, studies have shown that the relative abundance of *Oscillospria* in patients with inflammation is significantly reduced, while the relative abundance of *Lactobacillus* and *Bifidobacterium* in the intestines of diabetic patients treated with acarbose increases (Konikoff and Gophna, 2016; Gu et al., 2017). And the probiotic compound of *Lactobacillus* and *Bifidobacterium* can relieve obesity and inflammation in rats induced by high-fat diet (Shin et al., 2018). Other studies have shown that *Akkermansia muciniphila* and *Acinetobacter calcoaceticus* significantly increase in patients with multiple sclerosis, and induce inflammatory responses in human peripheral blood mononuclear cells and mice (Cekanaviciute et al., 2017). Therefore, the increase in the abundance of the beneficial intestinal bacteria *Lactobacillus*, *Bifidobacterium* and *Oscillospria*, and the decrease in the abundance of the conditional pathogen *Acinetobacter* confirmed that the BefA protein can improve the symptoms of diabetic mice by regulating the intestinal microbiota.

In summary, our results show that BefA protein reversed blood sugar, improved glucose tolerance, and maintained body weight in T1DM mice, *via* improving intestinal permeability, inhibiting intestinal inflammation, increasing intestinal beneficial bacteria *Lactobacillus*, *Bifidobacterium*, *Oscillospria* and reducing the relative abundance of conditional pathogenic bacteria *Acinetobacter*. In this study, we first explored the therapeutic effect of BefA protein on T1DM mice induced by MLD-STZ injections and the potential mechanism of intestinal microbiota in the treatment, which provides data support for its potential clinical application.

## Data Availability Statement

The datasets presented in this study can be found in online repositories. The names of the repository/repositories and accession number(s) can be found in the article/supplementary material.

## Ethics Statement

The animal study was reviewed and approved by Laboratory Animal Ethics Committee of Nanchang Royo Biotech Co,. Ltd.

## Author Contributions

TC contributed to conception and design of the study. HH, QQ, JW, XZ, and FL performed the experiments. HH, QQ, YC, and YL carried out data analysis. All authors contributed to the article and approved the submitted version.

## Funding

This study was supported by the National Natural Science Foundation of China (Grant no. 82060638), Academic and technical leaders of major disciplines in Jiangxi Province (Grant no. 20194BCJ22032), and Double thousand plan of Jiangxi Province (high end Talents Project of scientific and technological innovation).

## Conflict of Interest

Author QQ was employed by company Harbin Meihua Biotechnology Co., Ltd.

The remaining authors declare that the research was conducted in the absence of any commercial or financial relationships that could be construed as a potential conflict of interest.

## Publisher’s Note

All claims expressed in this article are solely those of the authors and do not necessarily represent those of their affiliated organizations, or those of the publisher, the editors and the reviewers. Any product that may be evaluated in this article, or claim that may be made by its manufacturer, is not guaranteed or endorsed by the publisher.
